# CO_2_ Fertilization in Plants from Physiological Mechanisms to Sustainable Deployment

**DOI:** 10.3390/plants15193016

**Published:** 2026-10-01

**Authors:** Yeding Xia, Lixiang Liu, Weiwei Liu, Tengfei Li, Bingran Ma

**Affiliations:** 1 State Key Laboratory of Environmental Criteria and Risk Assessment/State Environmental Protection Key Laboratory of Regional Eco-Process and Function Assessment, Chinese Research Academy of Environmental Sciences, Beijing 100012, China; xiayd@craes.org.cn (Y.X.); liuww@craes.org.cn (W.L.); ma.bingran@craes.org.cn (B.M.); 2 State Key Laboratory of Earth Surface Processes and Disaster Risk Reduction, Zhuhai Branch, Beijing Normal University at Zhuhai, Zhuhai 519087, China; 202431051115@mail.bnu.edu.cn; 3 Guangdong-Hong Kong Joint Laboratory for Carbon Neutrality, Jiangmen Laboratory of Carbon Science and Technology, Jiangmen 529199, China

**Keywords:** elevated CO_2_, photosynthetic acclimation, source–sink relations, nutrient limitation, crop quality, controlled-environment agriculture

## Abstract

Rising atmospheric carbon dioxide (CO_2_) enhances C_3_ photosynthesis and often improves intrinsic water-use efficiency, yet the extent to which this physiological potential translates into durable agronomic and ecological benefits remains uncertain. This review integrates evidence from controlled environments, free-air CO_2_ enrichment experiments, field studies, and modelling to evaluate the mechanisms, constraints, and applications of CO_2_ fertilization. We synthesize this evidence within a cascade-filtering framework that follows carbon gain from leaf photosynthesis through whole-plant allocation, resource limitation, environmental stress, and belowground processes to realized outcomes. Three major conclusions emerge from this review. First, leaf-level photosynthetic gains are progressively attenuated by photosynthetic acclimation, source–sink limitation, nutrient shortage, and water and heat stress, so biomass and harvestable-yield responses are smaller and more variable. Second, yield gains can coincide with lower protein, iron, and zinc concentrations, making nutrient yield as important as mass yield. Third, greater carbon input belowground does not guarantee persistent soil-carbon storage because allocation, turnover, and rhizosphere priming determine retention. In controlled-environment agriculture, enrichment is most effective when CO_2_ supply is coordinated with light, climate, water, nutrients, crop load, energy use, and gas delivery. Future progress requires multi-factor, multi-year, genotype-resolved studies that link physiology with nutritional quality, soil processes, modelling, life-cycle impacts, and economics.

## 1. Introduction

The term CO_2_ fertilization is commonly used for the increase in plant carbon assimilation that follows a rise in the CO_2_ concentration around photosynthetic tissues [1,2]. The expression is useful, although it can be misleading when it suggests a fertilizer-like response with a predictable dose effect. CO_2_ supplies carbon for photosynthesis and also alters stomatal behaviour, but conversion of newly fixed carbon into new tissues depends on several linked processes. Mineral nutrition supplies N, P, K, and micronutrients that are essential for the synthesis of proteins and nucleic acids, energy metabolism, enzyme activity, osmotic regulation, and the development of new tissues [3,4,5,6,7]. Hydraulic function maintains plant water status, cell turgor, stomatal regulation, and the pressure gradients required for long-distance assimilate transport [1,3]. Photoassimilates mainly in the form of sucrose are transported through the phloem from source leaves to metabolically active sink organs, including meristems, roots, developing grains, fruits, and storage tissues [8,9,10]. The capacity of these sinks to import, metabolize, and store assimilated carbon ultimately determines whether the additional carbon fixed under elevated CO_2_ is converted into structural biomass or harvestable yield [8,9,10,11]. When nutrient supply, hydraulic transport, phloem loading and unloading, or sink activity becomes limiting, carbohydrates can accumulate in source leaves, thereby triggering feedback inhibition and photosynthetic acclimation, including reductions in Rubisco abundance, leaf N, and photosynthetic capacity [9,10,11,12]. Variation in these processes is already evident before differences in biomass or yield are measured.

Three related bodies of literature have developed around this response. Global-change studies examine crops, forests, and grasslands under the continuing rise in atmospheric CO_2_, and free-air CO_2_ enrichment (FACE) experiments have been central to testing these responses under open-air conditions [13,14,15]. Controlled experiments use chambers and open-top systems to isolate mechanisms, whereas commercial enrichment treats CO_2_ as a production input in greenhouses and plant factories [16]. The concentration applied, duration of exposure, air mixing, rooting environment, and intensity of management differ substantially among these settings. A positive response in a chamber therefore cannot be assumed to represent the size of the field-yield response or the economic value of enrichment.

To distinguish these scenarios consistently, we use elevated CO_2_ for any atmospheric or experimental CO_2_ concentration above its stated ambient or control condition, CO_2_ enrichment for the deliberate addition of CO_2_ in experimental or commercial systems, and CO_2_ fertilization for the biological response caused by greater CO_2_ availability rather than for the treatment itself [1,2,13,16].

A second source of disagreement is the endpoint used to define benefit. Leaf assimilation can respond within minutes, while biomass, harvestable yield, and nutrient composition integrate acclimation and allocation over much longer periods. Intrinsic water-use efficiency does not describe seasonal evapotranspiration, and temporary incorporation of CO_2_ into plant biomass does not establish persistent carbon storage [17,18]. Systems-level analyses have emphasized that yield improvement requires coordination between carbon supply, sink development, and resource acquisition [8]. These distinctions are important because conclusions that appear contradictory often concern different biological scales.

We therefore organize the evidence as a cascade with five sequential filters. Stage 1 is the immediate biochemical and stomatal response that determines potential carbon gain. Stage 2 is whole-plant acclimation and source–sink use. Stage 3 comprises nutrient, water, heat, vapour-pressure deficit, ozone, and other resource and environmental constraints. Stage 4 tracks belowground allocation, turnover, and persistence through time and space. Stage 5 represents management and production-system constraints that determine agronomic, ecological, and economic outcomes. The cascade is not a fixed mass balance because the strength of each filter depends on species, genotype, CO_2_ exposure, environment, and the endpoint being evaluated.

**Figure 1:**
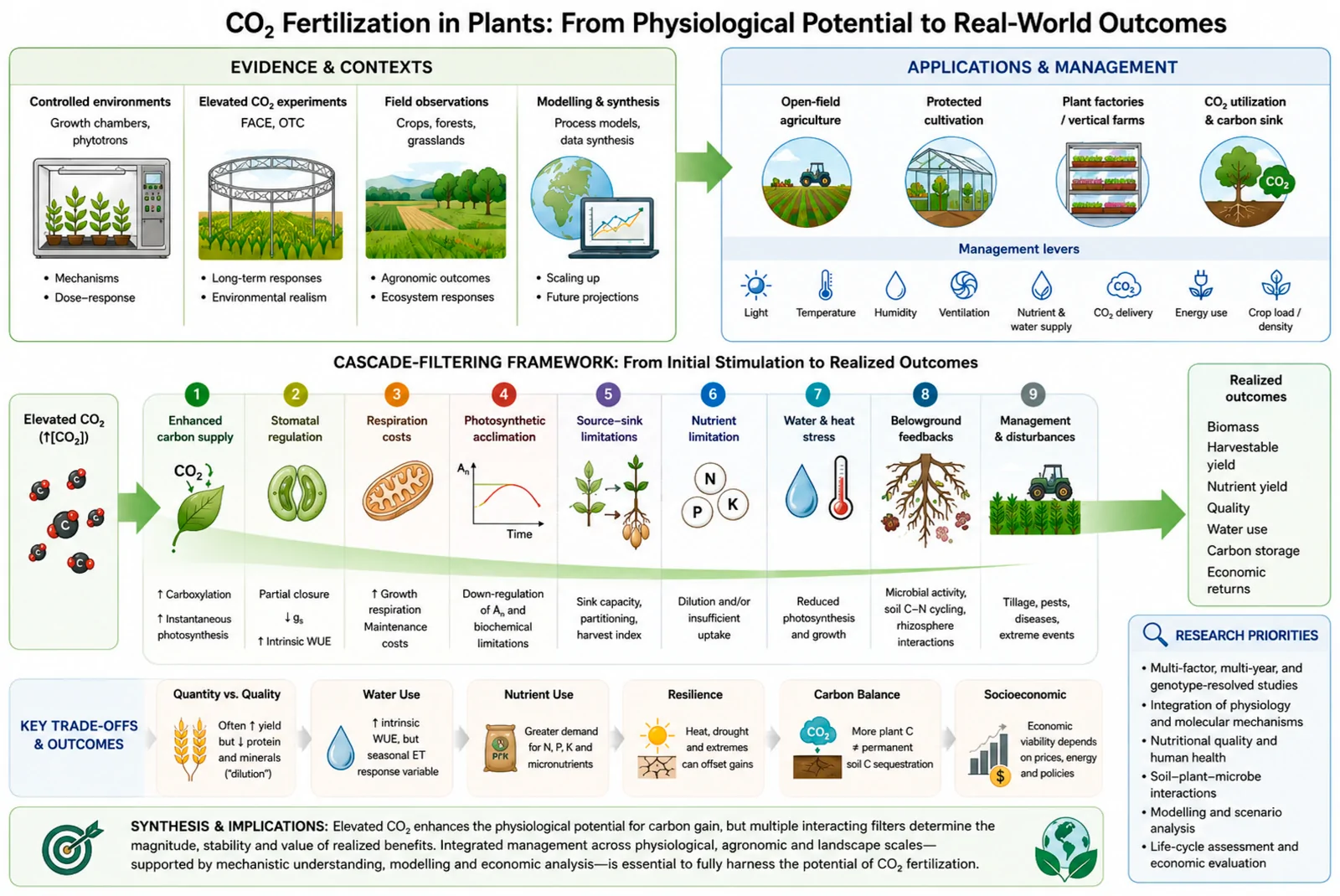
Conceptual overview linking elevated CO_2_ with physiological responses, constraints, and realized plant and production outcomes. The cascade is conceptual rather than a fixed quantitative mass balance because stage-specific attenuation varies among crops, environments, and endpoints. Upward (↑) and downward (↓) arrows indicate increases and decreases, respectively, under elevated CO_2_ relative to ambient CO_2_ conditions; they do not denote statistical significance. Green connecting arrows indicate conceptual links and progression from evidence to applications and from physiological stimulation to realized outcomes. The tapering green band illustrates the potential attenuation of initial physiological gains by interacting constraints and does not represent a quantitative reduction. g_s_, stomatal conductance; WUE, water-use efficiency.

Three key messages structure the review. First, CO_2_ fertilization represents physiological potential rather than a guaranteed increase in harvestable yield or persistent carbon storage, and Section 3, Section 4 and Section 5 examine where that potential is reduced or redirected. Second, benefit must be evaluated at the relevant endpoint, including harvestable yield, nutrient output, seasonal water use, and persistent carbon rather than leaf assimilation alone. Third, deliberate CO_2_ enrichment is sustainable only when biological gains are considered together with CO_2_ use, energy demand, water and nutrient efficiency, product quality, and economic performance, as examined in Section 6. Figure 1 summarizes this stepwise pathway from physiological stimulation to realized outcomes.

## 2. Experimental Approach to Studying CO_2_ Fertilization

### 2.1. Experimental Systems from Controlled Environments to FACE

Evidence for CO_2_ fertilization comes from platforms that answer different biological questions (Table 1). Leaf gas-exchange measurements resolve biochemical and stomatal responses with high temporal precision, but they say little about long-term sink limitation. Chambers and cabinets make factorial experiments possible, although enclosure can alter radiation, air movement, humidity, rooting conditions, and pest and disease pressure. FACE retains open-air canopy and soil conditions and has therefore become the main experimental basis for evaluating field-scale crop responses [12,14,19]. FACE provides realistic open-air conditions, but it also has important limitations because the achieved CO_2_ concentration can vary across space and time, while the high cost of the infrastructure usually limits the number of independent FACE experimental plots and study sites. The value of a study consequently depends on whether its experimental scale matches the inference being made.

Pot experiments illustrate particularly clearly how restricted rooting volume and altered resource availability can bias the apparent CO_2_ response. Passioura [20] showed why container studies can change plant water and nutrient relations, and a later meta-analysis by Poorter et al. [21] demonstrated that restricted rooting volume can strongly affect growth. Under elevated CO_2_, small pots can restrict sink development and accelerate feedback inhibition, while intensive fertilization and protected conditions may push plants toward an unrealistically resource-rich state. These effects can move the apparent CO_2_ response in opposite directions. Pot size per plant and rooting duration should therefore be treated as biological features of the experiment rather than as incidental details.

No experimental platform provides a complete hierarchy of truth. Controlled systems remain necessary for testing mechanisms, while field and commercial studies are required to establish agronomic relevance. FACE syntheses show the value of testing mechanisms under realistic competition and rooting conditions [12,19], whereas meta-analytic work demonstrates that experimental setting itself can explain part of the heterogeneity among studies [22,23]. The strongest conclusions are those that persist when biochemical measurements, longer-term experiments, field observations, crop, land-surface, and Earth-system models are considered together. For mechanistic causation, controlled gas-exchange and factorial experiments receive greater inferential weight, whereas estimates of field-yield magnitude are weighted toward FACE and multi-year field studies. Commercial studies are most informative for operational and economic outcomes but are less suited to isolate CO_2_ as a single causal driver.

**Table 1:** Experimental platforms used to study CO_2_ fertilization and their implications for inference.

Platform	Primary Inference	Strength	Limitation	Implication for Interpretation	References
Leaf gas exchange/A–Ci curves	Biochemical, stomatal, and mesophyll limitation	High temporal resolution and direct parameterization	Short exposure and weak connection to whole-plant sinks	Best used to identify mechanism rather than predict yield	[1,24]
Growth chamber/cabinet	Mechanisms and factorial interactions	Precise control of CO_2_, temperature, light, and water	Altered spectra, turbulence, humidity, and rooting environment	Treatment effects may include enclosure responses	[13,25,26]
Pot or container study	Genotypic contrasts and root traits	Efficient screening with destructive root and tissue sampling	Root restriction and rapid changes in water and nutrients	Pot volume can alter sink strength and acclimation	[20,21]
Open-top chamber	Semi-field acclimation and crop response	Natural soil with partial exposure to field conditions	Heating and boundary-layer effects	Microclimate differences need to be considered when comparing with ambient plots	[13,22]
FACE	Field-scale response under open air	Realistic canopy competition and rooting conditions	High cost, few sites, and variable achieved CO_2_	Strong basis for field inference but limited replication constrains generalization	[12,14,19]
Commercial greenhouse/plant factory	Operational yield and resource efficiency	Direct relevance to production management	CO_2_ response is confounded with light, ventilation, heating, and crop load	Agronomic response should be interpreted together with gas and energy use	[16,27,28]
Remote sensing/flux observations	Regional greening, WUE, and carbon uptake	Broad spatial and temporal coverage	Attribution is indirect because drivers covary	Most useful when combined with experiments and independent attribution	[18,29]
Crop/land-surface models	Scenario projection and scaling	Integrates processes across time and space	Structural uncertainty and parameter compensation	Model credibility depends on evaluation against field experiments	[30,31,32,33,34,35]

### 2.2. Methodological Factors Shaping CO_2_ Responses

Because conclusions can shift when incompatible experimental contexts are pooled, evidence selection and statistical dependence must be explicit. We use the reporting principles of PRISMA 2020 and ROSES to document study selection [36,37], while recognizing chambers, FACE plots, field plots, cultivars, and years as potentially non-independent levels when interpreting quantitative syntheses [38,39,40] (Table 2). These frameworks are also useful for critical reviews because they make the literature search and study-selection process transparent and help readers assess the evidence supporting the conclusions. A robust synthesis should first identify how experimental design, plant material, resource supply, and exposure conditions modify the CO_2_ response, and only then determine whether a single overall effect size is biologically meaningful. Accordingly, CO_2_ concentration and duration, photosynthetic pathway, genotype, developmental stage, rooting volume, light, temperature, vapour-pressure deficit, water supply, and N, P, and K availability are treated as biological moderators rather than pooled indiscriminately [23].

Primary experiments are most informative when achieved CO_2_ exposure is reported together with independent replication, microclimate, rooting conditions, nutrient and water supply, plant density, cultivar, and phenology. For commercial greenhouse studies, gas consumption, leakage, source purity, energy use, crop quality, and economic performance become part of the experimental context. These variables determine whether two nominally similar CO_2_ treatments are biologically comparable. Their omission is one reason why statistically clear results can remain difficult to interpret across studies.

## 3. Physiological Mechanisms and Crop Performance

Within the cascade, Section 3 addresses Stages 1 and 2 by following the immediate biochemical and stomatal response into acclimation, respiration, source–sink regulation, and yield formation (Table 3).

### 3.1. Physiological and Biochemical Responses

In C_3_ leaves, elevated CO_2_ increases chloroplastic CO_2_ availability and shifts Rubisco toward carboxylation rather than oxygenation. The Farquhar-von Caemmerer-Berry model formalized how assimilation changes when limitation lies with Rubisco activity, ribulose-1,5-bisphosphate regeneration, or triose-phosphate utilization [24]. Short-term stimulation is greatest when light and temperature allow the additional carbon to be used, and moderate warming can increase the relative advantage because photorespiratory losses are larger at ambient CO_2_ [13,25,26]. This biochemical potential represents the beginning of the response rather than its final agronomic expression.

C_4_ species respond differently because their CO_2_-concentrating mechanism already raises CO_2_ around Rubisco. Direct photosynthetic stimulation is therefore small under well-watered conditions. Maize FACE studies nevertheless showed that elevated CO_2_ can become beneficial during drought because lower stomatal conductance conserves soil water [91,92]. CAM species depart further from this pattern because nocturnal uptake and internal carbon storage alter the periods in which external CO_2_ constrains assimilation. The photosynthetic pathway is thus important, but it does not by itself predict the crop response under field stress.

Stomatal conductance generally declines as CO_2_ rises because stomatal pores partially close, reducing aperture and transpiration. With longer exposure, elevated CO_2_ can also modify stomatal development, most commonly by reducing stomatal density, while changes in stomatal size are more species- and environment-dependent [1,53,54,93]. The immediate result is lower transpiration per unit leaf area and a marked increase in intrinsic water-use efficiency. At the crop scale, seasonal water use may decline much less than leaf-level transpiration because elevated CO_2_ can increase leaf area and prolong canopy duration. The resulting increase in evaporative surface area, together with warmer leaves caused by reduced evaporative cooling, can partly offset the water savings from lower stomatal conductance. The water response therefore depends on whether it is measured at the leaf, canopy, or seasonal scale.

Respiration provides a counter-flux that is less consistent than photosynthesis. Respiration per unit tissue mass can decline when tissue N or maintenance demand falls, but total plant respiration may increase as biomass accumulates and more carbon is allocated belowground. Temperature adds another layer because respiratory carbon loss rises rapidly under warm conditions. Work in rice has shown that respiratory costs can be more closely related to yield loss under moderate heat than photosynthesis itself [94], and broader syntheses reach a similar conclusion for the balance between photosynthetic gain and respiratory demand [25]. Carbon-use efficiency therefore cannot be inferred from assimilation alone.

When plants are exposed to elevated CO_2_ for weeks to months, the initial stimulation of photosynthesis often becomes smaller than the response measured immediately after CO_2_ is increased. This long-term adjustment, termed photosynthetic acclimation, is commonly associated with lower Rubisco content, reduced maximum Rubisco carboxylation capacity (Vcmax), lower leaf N, and changes in the expression of photosynthesis-related genes [9,10,11,12]. In some plants, these changes represent an efficient reallocation of N and energy away from carboxylation capacity that is no longer fully required at high CO_2_. Acclimation becomes a stronger limitation when extra carbohydrates accumulate because phloem export, root growth, reproductive sinks, or nutrient supply cannot use the additional carbon rapidly enough. Thus, similar reductions in Vcmax or Rubisco can reflect either adaptive resource reallocation or insufficient sink and nutrient capacity, and these mechanisms should be distinguished when interpreting long-term CO_2_ responses.

Source activity and sink demand are linked from the biochemical to the developmental scale. Triose-phosphate utilization can constrain assimilation within minutes, while sucrose signalling, trehalose-6-phosphate, N status, leaf age, and developmental phase alter source activity over days and weeks. Systems-level analyses of crop improvement have consequently questioned strategies that target leaf photosynthesis in isolation [8]. Additional carbon contributes to yield only when it is transported to growing meristems and reproductive sinks and retained in organs of economic value.

**Table 3:** Physiological processes linking elevated CO_2_ with plant and crop outcomes.

Process	Initial Response	Counter-Process	Measurements	Representative Crop/Plant System	References
Rubisco carboxylation and photorespiration	Higher carboxylation and lower photorespiration in C_3_ leaves	Lower Vcmax or Rubisco under sink or N limitation	A–Ci curves, Vcmax, Jmax, Rubisco content	C_3_ crops and model C_3_ species (e.g., wheat, soybean, rice)	[1,24,25]
Stomatal regulation and transpiration	Lower stomatal conductance and higher intrinsic WUE	Canopy expansion and warming can offset leaf-level water saving	gs, transpiration, leaf temperature, ET	multiple terrestrial crops and species	[1,53,54]
Respiration and carbon-use efficiency	Potentially greater net carbon gain at tissue scale	Larger biomass and heat can increase total respiratory loss	Leaf, root, and ecosystem respiration	rice and mixed C_3_ species	[25,94]
Carbon export and source feedback	Greater sugar and starch production	Carbohydrate accumulation can inhibit further photosynthesis	Sugars, starch, TPU, phloem transport	soybean and other C_3_ crops	[9,10,11]
Sink development and yield formation	Potentially more reproductive or storage organs	Sink number or filling capacity may saturate or become heat sensitive	Grain number, fruit load, harvest index	major grain and seed crops, including rice and soybean	[14,41,52]
Nutrient acquisition and quality	Potentially greater root growth and uptake	N, P, K, Fe, Zn, or nitrate assimilation can constrain the response	Ionome, uptake flux, nutrient yield	wheat, Arabidopsis, rice, and multi-crop/global syntheses	[5,6,7,55]

### 3.2. From Carbon Assimilation to Growth and Yield Through Source–Sink Regulation

Biomass integrates carbon gain across canopy area and time and then subtracts respiratory loss before allocation among organs. Elevated CO_2_ often increases leaf area, branching, tillering, root growth, and total dry matter. Reproduction adds a different set of constraints through flowering, pollen viability, fertilization, seed or fruit set, filling rate, and harvest index. A meta-analysis of reproductive responses found a positive mean effect but substantial variation among species and reproductive traits [52,95]. The magnitude of vegetative growth is therefore a poor predictor of the amount of additional carbon ultimately allocated to the harvested organ.

The gap between physiological potential and harvestable yield became particularly clear with FACE. Long et al. [14] reported that yield stimulation under open-air enrichment was smaller than estimates derived largely from enclosure studies. Three decades of FACE research have since confirmed a genuine fertilization effect while also showing that field competition, variable CO_2_, realistic rooting, pests, disease, and resource limitation reduce the response expected from leaf physiology [19]. This difference does not represent a failure of the CO_2_ mechanism but rather reflects additional processes and interactions that emerge at larger scales of observation.

Apparent disagreement between chamber and FACE estimates should therefore not be treated as conflicting evidence of equal relevance. Enclosures provide strong mechanistic control but can alter rooting volume, boundary layers, pest exposure, and resource supply, whereas FACE preserves field competition and multiple stresses at the cost of lower replication and more variable achieved CO_2_ [12,14,19,22,23]. For estimating the magnitude of field-yield responses, FACE and multi-year field evidence therefore carry greater weight, while chamber studies remain more informative for isolating individual mechanisms.

Genotypic variation provides direct evidence for the importance of source–sink balance. Soybean genotypes differ substantially in their ability to sustain photosynthesis and convert additional carbon into seed yield under FACE [11]. In rice, the high-yielding cultivar Takanari maintained strong sink activity and avoided part of the N-associated down-regulation seen in more sink-limited material [41]. Potato cultivars also differed in photosynthesis, water-use efficiency, tuber initiation, and final yield response [42]. These comparisons point toward sink number, sink size, phloem transport, remobilization, and nutrient capture as breeding targets that complement photosynthetic capacity.

A large vegetative response can even reduce agronomic value when carbon accumulates in leaves and stems rather than harvestable organs. Excessive vegetative growth may delay maturity, alter root-to-shoot allocation, weaken structural tissues, or lower reproductive allocation. High carbohydrate status can also shift tissue C-to-N balance and secondary metabolism, with consequences for defence and trophic interactions. Such responses are informative because they reveal where carbon acquisition becomes disconnected from crop value.

### 3.3. Species, Genotypic, and Developmental Variation

Average effect sizes conceal biologically important differences among functional groups. C_3_ crops generally show a stronger direct photosynthetic response than C_4_ crops [3], but drought, temperature, and developmental stage can change the ranking observed at harvest. Legumes can partly relax N limitation through symbiotic fixation, although the benefit depends on P supply, water availability, and reproductive demand. Perennial species allocate carbon among woody biomass, storage reserves, roots, defence mechanisms, and symbiotic associations over multiple years, and long-term experiments indicate that these carbon-allocation patterns can shift dynamically over time [96,97]. Annual crops have a different constraint because their value depends on rapid transfer to harvestable organs within a single season.

Variation within a species is equally important. Bishop et al. [11] found large differences among eighteen soybean genotypes in photosynthetic and yield responses under FACE. Comparable contrasts have been reported for the rice cultivar Takanari and for potato cultivars that differ in water-use efficiency and tuber yield [41,42]. This variation creates opportunities for breeding, but it also means that results from one or two cultivars cannot be generalized to an entire crop.

Developmental stage determines when the apparent CO_2_ benefit is tested. Seedling experiments emphasize vegetative growth, whereas final yield can be set much later by reproductive heat stress, drought, nutrient shortage, or failure of grain and fruit set. Maize provides a useful example because elevated CO_2_ can support reproductive performance under drought even when C_4_ photosynthesis changes little under ample water [91,98]. Experiments that end before flowering therefore miss a large part of the biology that determines harvestable yield.

Breeding for future CO_2_ environments will require coordinated traits rather than a single photosynthetic target. Sink strength, phloem transport, root acquisition of N, P, K, Fe, and Zn, retention of grain protein, and reproductive heat tolerance all influence the fate of additional carbon [8,99]. High-throughput phenotyping is most useful when these traits are measured together with canopy carbon gain and nutrient output. Selecting genotypes solely for higher single-leaf assimilation may improve the initial carbon-fixation step without removing downstream constraints on assimilate transport, sink capacity, nutrient acquisition, and reproductive yield.

## 4. Environmental Constraints, Nutritional Quality, and Belowground Feedbacks

Section 4 examines Stage 3 and the belowground component of Stage 4, where resource supply, water and heat stress, atmospheric demand, ozone, food-quality changes, and rhizosphere processes determine how much of the initial carbon gain remains agronomically or ecologically valuable (Table 4).

### 4.1. Environmental and Resource Constraints

Nitrogen has received the most attention because key photosynthetic proteins and enzymes, such as ribulose-1,5-bisphosphate carboxylase/oxygenase (Rubisco), Rubisco activase, ATP synthase, and nitrate reductase, nucleic acids, and new sink tissues all require N. Elevated CO_2_ often lowers tissue N concentration through biomass dilution, changes in transpiration-driven mass flow, altered root uptake, and reduced nitrate assimilation. Bloom et al. [5] showed that shoot nitrate assimilation can be inhibited under elevated CO_2_ in some C_3_ species. Long-term experiments further show that productivity can become progressively constrained when additional carbon input is not matched by N availability [43,44]. Nitrogen limitation is therefore both a physiological and a temporal process.

Increasing N supply can strengthen the CO_2_ response when N is limiting, but the effect does not continue indefinitely. Once the immediate limitation is relieved, further N input can lower nitrogen-use efficiency and increase nitrate loss and N_2_O emissions. Legumes partly avoid this dependence through biological N fixation, yet fixation itself varies with P supply, water status, nodulation, and the carbon cost of maintaining symbionts [45]. The objective is therefore not simply to apply more fertilizer, but to supply nutrients in proportion to the additional growth and sink demand created by elevated CO_2_. Once one nutrient limitation is relieved, another resource can become limiting.

Phosphorus can become the dominant constraint in systems where additional carbon demand is not matched by P acquisition. P is required for ATP production, nucleic acids, membranes, sugar-phosphate metabolism, and symbiotic nutrient acquisition. Meta-analysis and FACE studies show that mycorrhizal association and soil P availability can determine whether higher photosynthesis is translated into biomass [46,47,48]. In low-P systems, carbon may be redirected toward roots and symbionts instead of aboveground growth. The result is sustained carbon uptake without a proportional increase in harvestable or woody biomass.

Potassium is discussed less often than N or P, although its functions make it directly relevant to CO_2_ responses. K regulates osmotic adjustment, stomatal movement, enzyme activity, phloem loading, and assimilate transport. A recent global synthesis indicates that K limitation is widespread enough to influence terrestrial carbon responses [7]. Micronutrients raise a related issue because reductions in Fe, Zn, Mg, or Mn are often first detected at harvest even though deficiencies can affect photosynthesis and reproduction earlier. Multi-element ionomics is therefore more informative than an N-only description of nutrient limitation.

The water benefit of elevated CO_2_ depends strongly on drought intensity and crop type. Lower stomatal conductance can delay soil drying in many C_3_ crops, but severe drought still imposes hydraulic limitation, metabolic inhibition, early senescence, and reproductive failure. Wheat and maize respond differently during progressive drought [49]. In soybean, intensifying drought can remove the expected CO_2_ benefit [50], whereas semi-arid wheat has shown substantial yield stimulation under some combinations of heat and water limitation [51]. Elevated CO_2_ can therefore buffer water stress without making crops drought-proof.

Temperature changes both the biochemical benefit and the biological cost of elevated CO_2_. Moderate warming can increase the relative C_3_ advantage because photorespiration rises under ambient CO_2_. At higher temperatures, respiration accelerates, phenological phases shorten, and pollen or grain filling can be damaged. Experiments and meta-analyses show that the net result depends on the balance between these processes [100,101,102,103]. Reduced transpiration can raise leaf temperature above the surrounding air temperature, particularly under strong radiation and high vapour-pressure deficit. However, CO_2_ × temperature studies do not identify a single temperature range that applies across crops because the leaf–air temperature difference is strongly species-, canopy-, and humidity-dependent [100,101,102,103]. For this reason, both air temperature and leaf or canopy temperature should be reported when interpreting CO_2_–temperature interactions.

Light and atmospheric evaporative demand, expressed as vapour-pressure deficit (VPD), determine whether additional CO_2_ can be used at a given moment. Low irradiance limits photochemistry and reduces the marginal return to enrichment, while high VPD can restrict stomatal opening even when CO_2_ is at ambient concentration and can also reduce the additional benefit obtained from elevated CO_2_. Ozone adds another stress interaction because it enters leaves primarily through stomata and promotes the formation of reactive oxygen species in the apoplast and mesophyll. These oxidative reactions can damage cell membranes and photosynthetic machinery, accelerate leaf senescence, and reduce biomass and yield [104,105,106]. Elevated CO_2_ often lowers stomatal conductance and can therefore reduce ozone uptake, but this partial protection does not eliminate oxidative injury under combined CO_2_–ozone stress. These interactions help explain why CO_2_ responses vary across seasons and canopy positions even within the same crop.

**Table 4:** Resource and environmental factors that modify the CO_2_ fertilization response.

Moderator	Conditions Associated with a Stronger Response	Conditions Associated with a Weaker Response	Key Endpoints	References
Nitrogen	Adequate N supports photosynthetic proteins and sink growth	Progressive N limitation and lower grain protein	N uptake, leaf N, grain N, nitrogen-use efficiency	[5,43,44]
Phosphorus	Adequate P supports adenosine triphosphate metabolism, root growth, and symbiotic acquisition	Low P restricts sink development and productivity	P fractions, root growth, phosphatase, mycorrhizae	[47,48,107]
Potassium	Adequate K supports stomatal regulation and assimilate transport	Low K weakens phloem loading and drought tolerance	Leaf K, stomatal conductance, harvest index	[7]
Water availability	Moderate water limitation can be buffered by slower soil drying	Severe drought causes hydraulic and reproductive failure	Soil water, evapotranspiration, water-use efficiency, yield	[49,50,51,98]
Temperature and heat	Moderate warming can increase the relative C_3_ biochemical benefit	Respiration, faster phenology, and reproductive damage reduce yield	Leaf temperature, respiration, fertility	[100,101,103]
Light and vapour-pressure deficit	High light supports use of additional CO_2_	Low light or high VPD lowers marginal return	Photosynthetic photon flux density, vapour-pressure deficit, canopy photosynthesis	[53,54,108]
Ozone and oxidative stress	Lower gs can reduce ozone uptake	Oxidative damage can still constrain yield	O_3_ flux, antioxidants, yield	[104,105,106]
Soil microbial feedbacks	Additional root C can stimulate nutrient mineralization and acquisition	Rhizosphere priming by fresh root-derived carbon can accelerate decomposition of existing soil organic matter and reduce long-term soil-carbon gain.	Rhizodeposition, soil organic carbon persistence	[46,69,70]

### 4.2. Nutritional Quality and Human Health

Yield alone is an incomplete measure of food production when edible-tissue composition changes at the same time (Table 5). FACE experiments in major C_3_ staples have shown that elevated CO_2_ can reduce the concentrations of iron (Fe) and zinc (Zn) in edible grain, with the magnitude varying among crops and cultivars [56]. In rice, elevated CO_2_ has additionally been associated with lower grain protein and reductions in several B vitamins, including vitamins B1, B2, B5 and B9, while responses of other compounds can differ in direction [57]. These results show that elevated CO_2_ can alter both mineral and organic components of nutritional quality rather than affecting a single nutrient class. Loladze [58] extended this pattern across a wider plant ionome and showed that mineral responses are not uniform among elements. Thus, elevated CO_2_ changes the nutrient composition of edible tissues, with some nutrients declining more than others, and not all nutrients being diluted to the same extent.

Several mechanisms can contribute to lower nutrient concentration. Faster carbohydrate accumulation can dilute nutrients. Reduced transpiration may limit the mass flow of certain mineral nutrients to the root surface, thereby restricting their subsequent uptake and translocation to the shoots. Elevated CO_2_ also changes root architecture, transporter activity, nitrate assimilation, remobilization, and allocation to seeds [5,59,60]. The relative importance of these processes varies with the plant species grown, the availability of nutrients in the soil, and the developmental stage of the plant. A single dilution mechanism cannot account for the range of responses observed across crops.

The health consequence depends on diet as much as on plant physiology. Models have projected greater risks of inadequate zinc and protein intake as atmospheric CO_2_ rises [61,62], and integrated analyses suggest that these nutritional changes can compound climate-driven effects on quality food availability [63]. Exposure is not evenly distributed because crop dependence, dietary diversity, baseline deficiency, and income vary among regions. Nutritional risk should therefore be evaluated within the broader context of the food system and dietary patterns through which a crop is consumed.

Crop assessment should report nutrient concentration and total nutrient yield together. If grain yield increases sufficiently, the additional grain mass can partly offset a decline in nutrient concentration; however, when the yield gain is small, total protein or mineral production per unit area may remain unchanged or may even decrease. Recent crop-focused syntheses show considerable variation among legumes, leafy vegetables, cultivars, and management regimes [55,64,65,66,67,68]. Breeding for nutrient retention, biofortification, balanced fertilization, and enhanced root nutrient acquisition have been proposed as potential strategies [4]. Their value will depend on whether they improve nutrient output without sacrificing yield stability.

The strength and direction of the quality response also differ among crop groups. In C_3_ cereals such as wheat and rice, evidence for lower grain protein and frequent reductions in Fe and Zn is comparatively consistent [56,57,59,67]. Legumes can partly buffer N limitation through symbiotic N fixation, but mineral responses remain sensitive to P supply, genotype, and soil nutrient availability [55,56,58]. Leafy and horticultural crops show more variable responses because nitrate, sugars, vitamins, and secondary metabolites depend strongly on light, N form, harvest stage, and controlled-environment conditions [64,65,66,67,68]. Thus, evidence for declining protein and mineral density is strongest for major C_3_ staple grains, whereas extrapolation to legumes and horticultural products requires more crop-specific interpretation.

### 4.3. Rhizosphere, Microbiome, and Soil-Carbon Processes

Elevated CO_2_ often increases the fraction of recent photosynthate transferred belowground through root growth, fine-root turnover, rhizodeposition, and exudation. These inputs stimulate microbial biomass, extracellular enzymes, mycorrhizal foraging, and nutrient mineralization. Fresh, labile carbon supplied through root exudates can stimulate microbial growth and extracellular-enzyme production. When this additional microbial activity increases demand for N or P, microorganisms may decompose pre-existing soil organic matter to acquire nutrients, thereby releasing additional CO_2_ from older soil carbon; this process is referred to as positive rhizosphere priming [70,71]. A larger carbon flux into roots and soil is therefore not equivalent to greater long-term soil-carbon storage. Carbon persistence depends on what happens after the new substrate enters the microbial and mineral soil pools. The direction and magnitude of this response also depend on carbon-to-nitrogen (C:N) stoichiometry. Carbon-rich inputs with a high C:N ratio can increase microbial N demand and promote decomposition of existing organic matter to acquire N, whereas lower-C:N substrates can alter the balance between microbial immobilization and net N mineralization. Consequently, greater carbon transfer belowground does not necessarily result in greater long-term soil-carbon storage; persistence depends on substrate quality, C:N balance, microbial processing and stabilization in mineral-associated pools.

Mycorrhizal strategy is one reason that belowground responses differ among ecosystems. Terrer et al. [46] showed that biomass responses to elevated CO_2_ vary with mycorrhizal association and N availability. The role of nutrient acquisition becomes especially clear in P-poor systems. At EucFACE, mature eucalypt woodland maintained enhanced photosynthetic carbon uptake without a corresponding increase in wood production [47]. Additional carbon was instead associated with stronger belowground activity, illustrating how nutrient acquisition can divert carbon away from long-lived aboveground pools.

Long-term FACE experiments also show that carbon fate depends on site history and resource supply. At Oak Ridge, productivity became constrained by N availability [43]. In mature forest, Jiang et al. [69] observed substantial allocation of additional carbon to fine roots and soil processes without a proportional increase in persistent woody carbon. Across experiments, greater plant carbon gain can coincide with reduced soil carbon storage when rhizosphere activity accelerates decomposition [70]. Net ecosystem carbon balance and persistence are therefore more informative endpoints than gross carbon input belowground.

Resolving this balance requires measurements that connect carbon input with carbon fate. Isotopic tracing can separate recent assimilates from older soil carbon, while root imaging and molecular analyses identify where new carbon enters biological pathways. Measurements of mineral-associated organic matter and greenhouse-gas fluxes are needed to determine whether additional input is stabilized or rapidly returned to the atmosphere. Models are beginning to represent root exudation and plant–microbe feedbacks more explicitly, although the persistence of these processes after CO_2_ enrichment ends remains poorly constrained.

## 5. Temporal and Spatial Scaling of Long-Term Responses and Modelling

Section 5 tests the persistence of Stages 2–4 across time and space by asking how acclimation, changing resource limitation, allocation, and turnover alter the response from leaves to canopies and ecosystems and from seasons to decades.

### 5.1. Time Scale, Acclimation, and Legacy Effects

CO_2_ responses unfold over time scales that range from minutes to decades. Gas exchange and stomatal behaviour respond rapidly, carbohydrate pools and gene expression adjust over hours and days, and leaf N, morphology, root allocation, and sink development change over weeks. Yield and quality integrate these adjustments over a season, while nutrient depletion, microbial change, and community turnover may require several years before they become evident. Exposure duration is therefore part of the biological treatment because different processes dominate at different stages of the response.

Short-term photosynthetic stimulation represents physiological capacity under a particular set of conditions. It does not forecast seasonal performance. FACE and long-term studies show that acclimation becomes stronger when N or sink capacity is limiting [12], while mature forests can maintain higher photosynthesis and still route much of the extra carbon into respiration, roots, symbionts, or turnover rather than wood [69]. The controlling process therefore changes with duration even when the direction of the leaf response remains positive.

Multi-year experiments further show that the identity of the limiting resource can change. Long-term grassland work has documented shifts in the relative responses of C_3_ and C_4_ species [96], and recent boreal evidence indicates that rising CO_2_ can reduce N availability over time [44]. Changes in rooting depth, community composition, and nutrient immobilization can all alter access to resources. A fertilization effect measured in the first years of an experiment should not be assumed to remain constant over a decade.

Much less is known about what remains after enrichment stops. Root systems, microbial communities, and nutrient pools may retain a legacy after leaf gas exchange has moved back toward ambient behaviour. Post-treatment measurements are needed to determine whether apparent carbon gains survive harvest, litter decomposition, product use, fire, and other turnover pathways. Without such observations, persistence is often assumed from carbon input rather than demonstrated from carbon retention.

### 5.2. Scaling from Leaves to the Globe and Model Prediction

Scaling from a leaf to a canopy or ecosystem requires more than multiplying a leaf-level percentage response by leaf area. Canopies contain strong gradients in light and N, new biomass carries construction and maintenance costs, and allocation changes with development. Stomatal responses also affect surface energy balance and atmospheric moisture, linking the CO_2_ response to the water cycle [18,29]. The same physiological mechanism can therefore produce different outcomes when it is embedded in canopy structure and ecosystem feedbacks.

Models differ in how they represent these processes, and this structural variation contributes substantially to projection uncertainty. Crop models translate CO_2_ effects into phenology, biomass, and yield, while land-surface and Earth-system models extend the response to regional and global carbon and water feedbacks. Intercomparisons show wide variation in projected wheat and global crop responses because photosynthesis, stomata, nutrient limitation, allocation, and soil carbon are represented differently [30,31,32]. FACE observations remain important because they test several of these processes simultaneously under open-air conditions.

Nutrient coupling is particularly influential in model projections. Carbon-only models usually produce a stronger fertilization response than models in which N or P limits growth. Evaluation of coupled carbon–nitrogen models against FACE data has shown that similar biomass responses can arise from compensating errors in uptake, allocation, and turnover [33]. Fixed allocation fractions create an additional problem when source–sink balance changes with development or resource supply [34,35]. Improving the process representation is therefore as important as calibrating the mean response.

Remote sensing offers an independent view of global greening and changes in water-use efficiency, but attribution remains difficult because CO_2_ rises together with climate change, N deposition, irrigation, land management, and recovery from disturbance. A synthesis of atmospheric constraints, inventories, remote sensing, experiments, and models provides stronger evidence for a CO_2_-driven terrestrial carbon sink than any single dataset [18]. Agreement among these lines of evidence is more informative than the magnitude estimated by one method alone.

## 6. Controlled-Environment CO_2_ Enrichment and Sustainable Deployment

Section 6 addresses Stage 5, where deliberate CO_2_ enrichment is filtered by crop management, gas delivery, ventilation, source purity, energy demand, economics, and life-cycle impacts (Table 6).

### 6.1. CO_2_ Enrichment in Controlled-Environment Agriculture

In greenhouses and plant factories, CO_2_ is a management input rather than an imposed climate scenario. Enrichment can increase canopy photosynthesis and marketable yield, but the useful concentration changes with light, temperature, VPD, ventilation, leaf area, crop stage, fruit or storage-organ load, and nutrient status. Earlier greenhouse research established these interactions [27,28], and recent reviews place them within a wider framework of controlled-environment management [16]. A single set-point used across changing conditions can therefore waste gas or favour vegetative growth without a proportional increase in marketable product.

Crop-specific studies show how strongly the response depends on the production environment. Greenhouse grape and strawberry experiments report gains in photosynthesis, yield, or fruit quality at selected CO_2_ concentrations [72,73], while cucumber studies demonstrate interactions with temperature, light intensity, relative humidity or VPD, water availability, and nutrient supply [74,108]. These results are difficult to compare when achieved canopy CO_2_, spatial uniformity, ventilation state, gas consumption, or enrichment duration are not reported. The biological response and the efficiency of CO_2_ delivery need to be considered together.

Evidence quality also differs across controlled-environment studies. Small chambers and tightly controlled facilities are well suited to isolate CO_2_ interactions with light, temperature, and humidity, but they can overstate operational efficiency when ventilation, leakage, and energy costs are minimized. Commercial greenhouse and plant-factory studies better represent marketable yield, gas use, and energy demand, although causal attribution is weaker because climate control, crop load, and management covary [16,27,28,72,73,74,75,76,77]. Biological responsiveness and production-system sustainability should therefore be evaluated with different endpoints rather than inferred from one another.

Control strategies are increasingly moving away from fixed timers toward photosynthesis-based prediction, sensor integration, and economic optimization. The aim is to match CO_2_ supply to the capacity of the canopy to use it under the prevailing light and climate. Model-predictive and digital approaches are developing rapidly [75,76,77], although validation across cultivars, seasons, and commercial growing structures remains limited. Their practical value will depend on whether improved control reduces gas loss and energy demand while maintaining crop quality.

CO_2_ source quality, leakage, and worker safety set practical boundaries around agronomic optimization. Food-grade liquid CO_2_ provides consistent purity but adds cost and embodied energy. Combustion-derived or industrial streams may be less expensive, although contaminants such as CO, NOx, SO_2_, ethylene, and particulates require control before the gas is used around crops and workers. Reliable enrichment also depends on calibrated sensors, adequate ventilation, and alarm procedures. These operational features influence both crop performance and the environmental balance of the production system. Fermentation of crop residues and other organic by-products can also generate a CO_2_-rich off-gas that may be recovered for greenhouse enrichment, creating a potential link between residue management and local CO_2_ reuse. However, the composition and continuity of the gas stream, the presence of methane or volatile contaminants, purification requirements, and the net life-cycle benefit must be evaluated before such systems are used around crops and workers. This option is therefore best assessed together with source purity, CO_2_-use efficiency and the fate of the residual biomass.

### 6.2. Economic, Energy, and Life-Cycle Sustainability

The economic return from CO_2_ enrichment is determined by the value of additional marketable yield and quality relative to the cost of gas supply, distribution, monitoring, and associated climate control. Greenhouse profitability therefore varies with crop price, season, enclosure tightness, lighting, energy tariffs, and the fraction of extra biomass that can actually be sold [78,79]. Because a percentage increase in yield alone does not indicate profitability, economic evaluation should be conducted at the scale of the whole production system by comparing the value of additional marketable yield and quality with the costs of CO_2_ supply, distribution, monitoring, ventilation and associated climate control.

Environmental performance follows the same logic. Plant factories can achieve high land- and water-use efficiency, but electricity for lighting and climate control may dominate the life-cycle footprint. Comparative studies of greenhouses and vertical farms show large effects of energy mix, building design, crop choice, and local climate [80,81,82,83]. Recent analyses also warn that expansion of indoor production without low-carbon electricity and efficient system design could increase energy-related impacts [75,84]. Local resource efficiency is therefore not equivalent to low greenhouse-gas emissions.

CO_2_ used to grow short-lived biomass should not be described as carbon removal unless the storage is demonstrably durable. Carbon incorporated into vegetables, grain, and crop residues is generally returned to the atmosphere through respiration, consumption, waste treatment, or decomposition. Life-cycle studies of CO_2_ utilization emphasize the need for an explicit counterfactual source, system boundary, energy demand, leakage, displacement effect, and storage duration [85,86,87]. Whether captured CO_2_ provides an avoided-emission benefit depends on the alternative source and the energy required to supply it.

Comparable evaluation across production systems requires a common set of biological, economic, and environmental endpoints. Marketable yield per unit area and time, product and nutrient output per unit supplied CO_2_, energy use per unit product, water- and nitrogen-use efficiency, economic return, and cradle-to-grave greenhouse-gas emissions have all been proposed for techno-economic and life-cycle assessment [88,89,90]. Considering these endpoints together distinguishes biological responsiveness from environmental sustainability. A CO_2_-enrichment system is more sustainable when yield and quality gains are achieved with high CO_2_-retention efficiency, low-carbon energy, efficient water and nutrient use, minimal leakage and greenhouse-gas emissions, safe gas quality, and an economic return that can be maintained over the production cycle.

## 7. Synthesis and Future Directions

### 7.1. Current Consensus and Unresolved Questions

The literature supports a direct physiological benefit of elevated CO_2_, but it does not support a simple expectation that this benefit will offset future climate damage. Warming, drought, ozone, nutrient limitation, and extreme events act on different stages of crop development and can erode the yield gain expected from carbon assimilation [4,14]. Response factors derived from chambers or historical climates should therefore be applied cautiously to warmer and more resource-limited futures. The uncertainty lies less in the existence of the photosynthetic response than in its persistence under interacting stresses.

The literature supports cumulative attenuation through the cascade but not a universal percentage loss that can be assigned to every stage. As an empirical benchmark, open-air FACE trials found crop-yield stimulation to be approximately 50% lower than estimates derived largely from enclosure studies [14]. This shows that a substantial part of the physiological potential is filtered before it appears as harvestable yield. However, the contribution of acclimation, source–sink limitation, nutrient supply, water and heat stress, and other filters cannot be uniquely partitioned because CO_2_ dose, crop, duration, environment, and endpoints differ among studies [12,19,23]. The uncertainty is even greater for persistent carbon pools. Forest and soil studies show that additional carbon uptake can be redirected to respiration, roots, microbial activity, and decomposition rather than retained in long-lived wood or stable soil organic matter [18,69,70]. We therefore interpret the cascade as a sequence of conditional filters, using harvestable yield for crop integration and persistent stock change, rather than gross uptake, for carbon sequestration. For illustration only, rather than as a universal quantitative rule, the cascade can be viewed as a sequence of conditional attenuation steps. The initial leaf-level CO_2_ response may be reduced progressively by acclimation and source–sink constraints, further constrained by nutrient, water, heat, and other environmental limitations, and ultimately filtered by allocation, turnover, and persistence processes. The approximate magnitude of attenuation at each stage will vary among crops, ecosystems, experimental conditions, and the endpoint considered. Therefore, these stages should not be interpreted as fixed conversion fractions. For crop production, yield is evaluated using empirical benchmarks such as the FACE versus enclosure comparison, whereas persistent carbon requires evidence of long-term stock change. No single conversion fraction from CO_2_ uptake to persistent carbon is defensible because carbon residence time depends on allocation, respiration, decomposition, microbial processing, disturbance, and stabilization pathways.

The water response is another case in which scale changes the conclusion. Intrinsic water-use efficiency usually rises because assimilation increases while stomatal conductance declines. Whole-season crop water use can remain similar when leaf area expands, or canopy temperature rises, as field and modelling studies have shown [29,49]. Claims that elevated CO_2_ reduces crop water requirement require evapotranspiration and yield-water productivity at the seasonal scale. Leaf gas exchange alone cannot establish that conclusion.

Nutrient supplementation can remove an immediate bottleneck without eliminating resource limitation. Additional N may strengthen growth in an N-limited system, yet it can shift limitation toward P, K, micronutrients, or symbiotic nutrient acquisition and can also increase environmental losses. Global analyses of N, P, and K limitation make this broader resource context increasingly clear [6,7]. The agronomic goal is coordinated nutrient supply that sustains sink development rather than restoration of an isolated photosynthetic response.

The ecosystem carbon question depends equally strongly on the endpoint considered. Enhanced photosynthesis and greening are well established, but long-term sequestration depends on allocation, tissue turnover, mortality, disturbance, and soil priming. Mature-forest experiments show that a substantial fraction of additional carbon can move through roots and microbial pathways without accumulating in long-lived wood or stable soil pools [18,69,70]. Greater photosynthetic carbon uptake can increase carbon inputs to biomass and soil, but it does not necessarily increase long-term carbon storage because the retained fraction is controlled by allocation, tissue turnover, decomposition, disturbance and soil stabilization processes.

Food-security implications also require the yield and quality responses to be considered together. Elevated CO_2_ can increase grain mass while reducing protein or mineral concentration [55,56,67]. Total nutritional output increases only when gains in edible biomass are large enough to offset any decline in nutrient concentration. The final outcome also depends on crop and genotype, nutrient supply, nutrient bioavailability, and the dietary context in which the crop is consumed. The evidence is more consistent with a context-dependent trade-off than with a uniformly positive or negative effect on food security.

### 7.2. Molecular Breeding and Crop Redesign Under Elevated CO_2_

Molecular breeding and gene editing offer ways to test several bottlenecks identified by elevated-CO_2_ studies, although most proposed targets remain to be validated under field CO_2_ enrichment. Stomatal development, Rubisco-related processes, Rubisco activase, mesophyll conductance, photorespiration, and source–sink regulation are all mechanistically relevant [4,8,99,109]. Their value will depend on whether changes at the leaf level improve whole-canopy carbon gain without compromising transpirational cooling, nutrient acquisition, or reproductive stability. Field validation is therefore essential before these targets can be considered adaptation strategies.

Traits that determine the fate of assimilated carbon may provide as much leverage as photosynthetic capacity. Sink strength, phloem loading, grain or fruit filling, root architecture, and mineral uptake determine whether additional carbon is retained in harvestable and nutrient-rich organs [8,99]. Genomic selection and targeted editing can be used to test these traits in combination rather than one at a time [109]. The useful breeding phenotype is a coordinated response of carbon assimilation, nutrient retention, and reproductive performance under elevated CO_2_.

### 7.3. Research Priorities

Future experiments need to resolve the conditions that generate contrasting CO_2_ responses rather than produce another average effect size. Multi-year designs should combine CO_2_ with warming, drought or high VPD, ozone, and multi-element nutrition while retaining sufficient genotypic replication to test sink traits and reproductive stability. Belowground studies need longer observations of carbon persistence, priming, and greenhouse-gas fluxes. Model development should proceed against FACE data so that acclimation, nutrient limitation, and dynamic allocation are constrained by experiments rather than adjusted only to match final biomass.

Controlled-environment agriculture provides a complementary setting in which CO_2_ can be manipulated as part of an integrated production system. Research should move beyond fixed concentration recipes and test control strategies that respond to light, ventilation, crop load, gas price, and energy demand. Across field and controlled systems, marketable yield, nutrient output, water and nutrient use, CO_2_ use, energy demand, greenhouse-gas balance, and uncertainty should be interpreted together. Such measurements would allow the field to identify when enrichment produces a durable benefit and when the initial physiological response is lost at later stages of the cascade.

## 8. Conclusions

Research over the last four decades has established a consistent leaf-level response to elevated CO_2_ in C_3_ plants, with higher photosynthetic carbon gain, lower stomatal conductance, and greater intrinsic water-use efficiency. The response becomes less predictable as it is followed into biomass, reproductive yield, food composition, and ecosystem carbon storage. Photosynthetic acclimation, source–sink relations, nutrient supply, water and heat stress, and belowground turnover all contribute to this divergence. CO_2_ fertilization is therefore best viewed as a conditional response whose magnitude depends on the scale and endpoint being measured.

Across crops and ecosystems, additional carbon is most likely to produce a durable benefit when sink capacity and nutrient acquisition keep pace with assimilation and when reproductive development is not constrained by heat or water stress. The same principle applies to controlled-environment agriculture, where crop response must be weighed against CO_2_ loss, energy use, product quality, and economic return. Future work should connect these processes rather than study them as separate responses. Doing so will provide a more realistic basis for predicting the agronomic, nutritional, ecological, and economic consequences of rising or deliberately enriched CO_2_.

## Figures and Tables

**Table 2:** Sources of methodological heterogeneity in CO_2_ studies and their consequences for interpretation.

Source of Heterogeneity	Why It Matters	Common Interpretive Consequence	References
Study context	Atmospheric rise, experimental enrichment, and commercial enrichment address different questions	Responses from ecological and production systems can be mixed as if they were equivalent	[2,13,16]
CO_2_ exposure	Achieved concentration, variability, timing, and duration determine dose and acclimation	Nominal set-points can obscure large differences in actual exposure	[12,13,19]
Experimental unit	Independent chambers, FACE rings, plots, and facilities define replication	Plant subsamples can be mistaken for independent treatment replicates	[12,14,19,38,39,40]
Plant material	Species, genotype, density, development, and sink status alter carbon use	Results from one cultivar or juvenile stage can be overgeneralized	[11,41,42]
Resource environment	Light, temperature, VPD, water, N, P, K, and rooting volume shape limitation	Responses may be attributed to CO_2_ when resource co-limitation is driving the difference	[3,6,7,43,44,45,46,47,48,49,50,51]
Response variable	Photosynthesis, biomass, harvestable yield, and quality occur at different scales	Leaf assimilation can be treated as a proxy for final crop performance	[8,14,52]
Water response	Intrinsic WUE and seasonal evapotranspiration describe different processes	Lower stomatal conductance can be interpreted as lower crop water requirement	[1,29,49,53,54]
Nutritional response	Concentration, total nutrient yield, and bioavailability answer different questions	Higher biomass can conceal lower nutrient density	[55,56,57,58,59,60,61,62,63,64,65,66,67,68]
Soil carbon	Inputs, respiration, stocks, persistence, and GHG fluxes determine net storage	Greater root input can be equated with sequestration	[46,69,70,71]
Controlled environments	CO_2_ use, leakage, energy, source purity, safety, and economics affect system performance	Temporary crop uptake can be interpreted as a climate benefit	[16,27,28,72,73,74,75,76,77,78,79,80,81,82,83,84,85,86,87,88,89,90]
Evidence synthesis	Dependence and experimental context affect pooled estimates	Studies from incompatible platforms can be combined without appropriate moderators	[23,36,37,38,39,40]

**Table 5:** Food-quality responses to elevated CO_2_, representative crops and major sources of variation.

Quality Trait	Frequently Reported Response	Candidate Mechanisms	Major Sources of Variation	Useful Endpoint	Representative Crop	References
Protein/grain N	Often lower in C_3_ cereals	Growth dilution, nitrate assimilation, remobilization, and genotype	N supply, legume fixation, tissue, and harvest stage	Concentration together with protein or N yield per unit area	Wheat and rice	[56,57,59,67]
Iron and zinc	Often lower in staple grains	Dilution, transpiration, uptake, and transport	Species, genotype, soil supply, and mycorrhizae	Concentration, bioavailability, and total Fe or Zn yield	Wheat, rice and other staple C_3_ grains/legumes	[56,57,58]
Carbohydrate, starch, and sugars	Often higher	Greater carbon assimilation and storage	Sink capacity, harvest maturity, and organ type	Dry-matter concentration and product quality	rice and horticultural crops	[57,64,65,66,67,68]
Vitamins and antioxidants	Mixed and compound-specific	Carbon–nutrient balance and secondary metabolism	Light, temperature, organ, and cultivar	Individual compounds rather than a single total antioxidant index	Rice and leafy/horticultural crops	[57,64,65,66,67,68]
Nitrate in leafy vegetables	Variable	N assimilation, growth rate, and light interaction	N form, light, harvest time, and species	Fresh- and dry-weight concentrations	kale, spinach and other leafy vegetables	[64,65,66,67,68]
Mineral ionome	Many elements decline, but responses are not uniform	Dilution, uptake, mass flow, and redistribution	Soil chemistry and element mobility	Multi-element profiles with quality-control materials	Multiple staple and edible plant species	[56,57,58,64,65,66,67,68]

**Table 6:** Factors governing the efficiency of CO_2_ enrichment in controlled environments.

Factor	Why It Changes the CO_2_ Response	Main Interaction	Useful Observations	Evaluation Endpoint	References
CO_2_ concentration	Marginal assimilation declines as CO_2_ rises	Crop stage and canopy sink demand	Canopy photosynthesis and achieved CO_2_	Marketable yield per unit supplied CO_2_	[16,27,28]
Enrichment timing	CO_2_ is most effective when the canopy is photosynthetically active	Light and photoperiod	PAR, photoperiod, and crop stage	CO_2_-use efficiency	[16,27,28,75,76,77]
Ventilation	Air exchange rapidly removes supplied CO_2_	Wind and indoor–outdoor gradients	Vent position, wind, and CO_2_ distribution	Retained CO_2_ fraction and economic return	[16,27,28,75,76,77]
Temperature and VPD	Both affect biochemistry, stomata, and leaf cooling	Heat stress and atmospheric demand	Leaf temperature, RH or VPD, and forecast	Assimilation, WUE, and reproductive performance	[27,28,74,108]
Water and nutrients	Additional carbon requires sink growth and mineral supply	Root-zone water, EC, and N, P, K supply	Water status, substrate EC, nutrients, and crop load	Marketable yield together with nutrient quality	[16,27,28,74,108]
CO_2_ source	Purity, cost, and embodied emissions differ among sources	Contaminants, worker exposure, and energy demand	Gas analysis, alarms, and life-cycle inventory	Net GHG balance and production cost	[78,85,86,87,88]

## Data Availability

No new data were created or analyzed in this study. Data sharing is not applicable to this review article.

## Abbreviations

CAM	Crassulacean acid metabolism
CEA	Controlled-environment agriculture
ET	Evapotranspiration
FACE	Free-air CO_2_ enrichment
LCA	Life-cycle assessment
N	Nitrogen
NUE	Nitrogen-use efficiency
OTC	Open-top chamber
P	Phosphorus
PAR	Photosynthetically active radiation
PPFD	Photosynthetic photon flux density
SOC	Soil organic carbon
TPU	Triose-phosphate utilization
VPD	Vapour-pressure deficit
WUE	Water-use efficiency
